# Multi-Scale Attention Feature Enhancement Network for Single Image Dehazing

**DOI:** 10.3390/s23198102

**Published:** 2023-09-27

**Authors:** Weida Dong, Chunyan Wang, Hao Sun, Yunjie Teng, Xiping Xu

**Affiliations:** 1 School of Opto-Electronic Engineering, Changchun University of Science and Technology, Changchun 130022, China; dwd@mails.cust.edu.cn (W.D.);; 2 Zhongshan Institute of Changchun University of Science and Technology, Zhongshan 528437, China

**Keywords:** image dehazing, image restoration, feature enhancement, color fidelity

## Abstract

Aiming to solve the problem of color distortion and loss of detail information in most dehazing algorithms, an end-to-end image dehazing network based on multi-scale feature enhancement is proposed. Firstly, the feature extraction enhancement module is used to capture the detailed information of hazy images and expand the receptive field. Secondly, the channel attention mechanism and pixel attention mechanism of the feature fusion enhancement module are used to dynamically adjust the weights of different channels and pixels. Thirdly, the context enhancement module is used to enhance the context semantic information, suppress redundant information, and obtain the haze density image with higher detail. Finally, our method removes haze, preserves image color, and ensures image details. The proposed method achieved a PSNR score of 33.74, SSIM scores of 0.9843 and LPIPS distance of 0.0040 on the SOTS-outdoor dataset. Compared with representative dehazing methods, it demonstrates better dehazing performance and proves the advantages of the proposed method on synthetic hazy images. Combined with dehazing experiments on real hazy images, the results show that our method can effectively improve dehazing performance while preserving more image details and achieving color fidelity.

## 1. Introduction

With the rapid development of modern industrialization and urbanization, haze has become a common natural phenomenon. Haze is composed of a large number of tiny particles such as water droplets and sulfur dioxide. When optical imaging equipment is collecting images, due to the scattering effect of these tiny particles, the contrast of the collected images is low, and many important details are lost. It is difficult to meet the requirements of object detection [1,2], target recognition [3,4], and other computer vision applications. Therefore, it is of great practical importance and application value to study the excellent performance of the dehazing algorithm to clarify the hazy images.

Single-image dehazing is a challenging problem, and many researchers have started to study single-image dehazing techniques [5,6,7,8]. The purpose of single image dehazing is to restore the image degraded by haze to a clear image. In recent years, most dehazing algorithms are based on a physical scattering model [9,10,11], which can be formulated as

(1) I(x)=t(x)J(x)+A(1−t(x))

where I(x) is the hazy image captured by the imaging device, J(x) is the restored haze-free image, A is the global atmospheric light, and t(x) is the transmission map. Unfortunately, in practical applications, both the transmission map and the atmospheric light are unknown. Therefore, most current image dehazing methods first estimate A and t(x), and then recover J(x) according to Equation (1).

These studies are based on physical models of atmospheric scattering and aim to restore images with prior knowledge. However, the optimal choice of prior knowledge is still unclear. In addition, the extent to which these priors obey the image statistics and how they influence the dehazing performance also remain unknown [12]. Recently, since the emergence of deep learning, learning-based methods have gradually been applied in the field of image dehazing [13,14]. Although existing learning-based methods have achieved remarkable success, Their performance is still limited by the loss of detailed information such as color [15].

Based on the above analysis, in order to avoid dehazing problems such as color distortion and incomplete dehazing, and to preserve as much image detail information as possible, an end-to-end single image dehazing network with multi-scale attention feature enhancement is proposed. This method can directly restore the input haze image to a clear image without estimating the parameters of the atmospheric scattering model.

The main contributions of this work are summarized as follows:

(1) We propose an end-to-end Multi-Scale Attention Feature Enhancement Networks for Single Image Dehazing (MAFE). This method has achieved excellent performance in image dehazing. It can adaptively focus on the high-frequency information of the hazy image and retain more detailed information. Since it does not need to rely on the atmospheric scattering model, it is not affected by the estimated atmospheric light value. Additionally, color distortion is avoided.
(2) We propose an attention feature enhancement module, which can adaptively focus on high-frequency information of hazy images, enhance the relevance of contextual information, suppress redundant information, and compensate for the loss of detailed information.
(3) We propose a multi-scale attention enhancement module that builds upon the attention feature enhancement module and incorporates a spatial pyramid of dilated convolutions to fully extract and utilize the multi-scale features of the image. This module expands the receptive field and improves the quality of the dehazed image while preserving more detailed information.
(4) The experimental results on both synthetic and real-world hazy images demonstrate that our proposed method achieves state-of-the-art single image dehazing methods in terms of dehazing performance. It can well preserve details such as color and texture of the image.

## 2. Related Work

Single image dehazing is an ill-posed problem. At present, the methods for single image dehazing are mainly divided into two categories, one is to artificially summarize the statistical difference between the blurred image and the unblurred image as an empirical prior, and the other is to directly or indirectly learn the mapping function from large-scale datasets of hazy and clear images. Researchers generally refer to the former as prior-based methods and the latter as learning-based methods.

The prior-based methods estimate the transmission rate and global atmospheric light based on some prior knowledge of the clean image. They usually rely on the atmospheric scattering model and handcrafted priors. He et al. [16] found that most local patches in outdoor haze-free images contain some pixels whose intensity is very low in at least one color channel, proposing dark channel priors (DCP). Zhu et al. [17] proposed a color attenuation prior by observing that the presence of haze can lead to image desaturation and brightness increase. Berman et al. [18] found that when haze appears, the pixel clusters of the haze-free image will become haze lines, and proposed a non-local prior to characterize the clean image. Fattal [19] proposes a method for estimating scene reflectance by assuming that the projection of the scene target surface and the propagation of light are partially uncorrelated to achieve dehazing images.

In recent years, with the rapid development of deep learning [20,21,22,23], many researchers have applied deep learning to image dehazing and designed a large number of dehazing neural networks. CAI et al. [24] proposed the Dehaze-net single-image dehazing network, which was the first to introduce convolutional neural networks into image dehazing tasks. It estimates the transmittance through operations such as multi-scale mapping and maximum pooling, and then clears the hazy image according to the atmospheric scattering model. Compared with traditional dehazing methods, Dehaze-net greatly improves the dehazing performance. Ren et al. [25] designed a coarse-scale network and a fine-scale network model to achieve dehazing by extracting and fusing the coarse transmission map and the fine transmission map. Li et al. [26] proposed All-in-One Network (AOD-Net), They unify the two parameters of atmospheric light value and transmittance in the atmospheric scattering model, convert them into a new variable, use lightweight convolution to estimate this variable and generate a dehazed image. Liao et al. [27] proposed HDP-net for night-time dehazing to restore haze-free images by estimating the haze density map. Chen et al. [28] proposed a gated aggregation network that uses dilated convolutions to increase the image receptive field, aggregates the semantic information of the image, and successfully solves the problem of network artifacts. Shao et al. [29] proposed a domain-adaptive dehazing network, which transforms images from one domain to another through a bidirectional transformation network to reduce the error between the synthetic domain and the real domain, and solve the problem of network artifacts. Qin et al. [30] proposed an end-to-end feature fusion attention network (FFA-net). They designed a novel feature attention module, which can effectively focus on the dense haze and high-frequency areas, and achieved excellent results in restoring synthetic hazy images. Liu et al. [31] proposed the GridDehazeNet dehazing network, which can generate learning inputs with better diversity and more relevant features. Through a novel attention-based multi-scale estimation, it effectively alleviates the bottleneck issue often encountered in the conventional multi-scale approach. Wang et al. [32] improved feature extraction and enhanced feature mapping, proposed a multi-scale supervision-guided context aggregation network (MSGCAN) based on two principles, and obtained better dehazing results. Based on the CIELAB color space, Sheng et al. [33] proposed a multi-scale residual attention network for single image dehazing, which improved the color performance of the dehazing method. Zhou et al. [34] proposed an attention-based feature fusion dehazing network, which uses attention-based residual dense blocks to enhance the details of low-light haze images.

## 3. Proposed Method

In this section, we first describe the haze density image prediction model and propose a haze density image prediction network based on multi-scale attention feature enhancement. Then, we introduce the dehazing fusion block (DFB), multi-scale attention module (MAE), attention feature enhancement module (AFE) and context enhancement module (CEM) in detail. Finally, we provide loss functions for training the network.

### 3.1. Haze Density Image Prediction Model

Since it is difficult to accurately estimate the atmospheric light value A in the atmospheric scattering model, HDP-net [27] redefines the atmospheric scattering model based on the formation principle of the fog map, which can be expressed as

(2) I(x)=J(x)+h(x)

where I(x) is the observed hazy image, J(x) is the real scene to be recovered, h(x) is the haze density image. At the same time, a haze density image prediction model is proposed as

(3) h(x)=N(I(x))

where N(⋅) is haze density image prediction network. Thus, the mathematical model of the haze-free image is obtained as

(4) J(x)=I(x)−N(I(x))=I(x)−h(x)

According to Equation (4), it is only necessary to build a haze density image prediction network N(⋅) to obtain the haze density image h(x), and then I(x) can restore to J(x). We propose a haze density image prediction network architecture with multi-scale attention feature enhancement. It can predict the haze density image h(x) in an end-to-end manner, thus restoring the haze-free image. Below, we will provide a detailed introduction to the multi-scale attention feature enhancement network architecture that we designed.

### 3.2. Network Architecture

**Figure 1:**
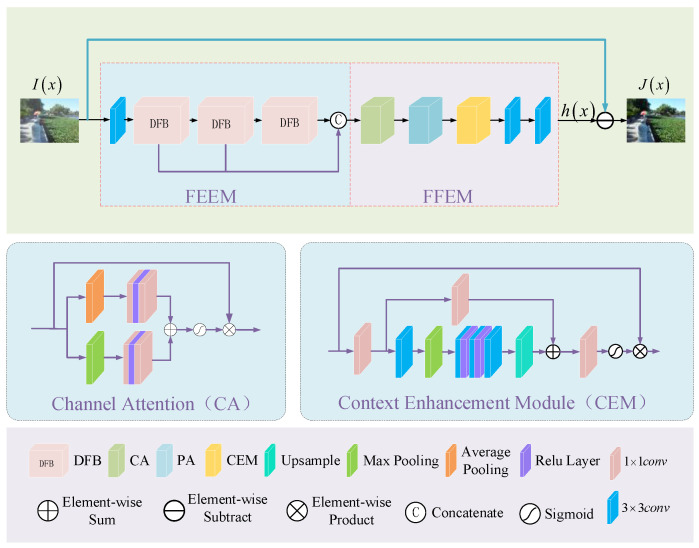
The architecture of the MAFE.

The multi-scale attention feature enhancement dehazing network (MAFE) we propose is shown in Figure 1. MAFE includes feature extraction enhancement module (FEEM) and feature fusion enhancement module (FFEM). FFEM is mainly composed of three dehazing fusion blocks (DFB), which are used to extract and fuse deep and shallow haze information. In DFB, we use the attention mechanism and dilated convolution, so the FEEM module has a larger receptive field and can capture more detailed information.

The FFEM mainly consists of channel attention (CA), pixel attention (PA) [30], context enhancement modules (CEM) [35], and two convolutional layers with a stride of 1.

The input of MAFE is the hazy image of the RGB channel. After it is transmitted to FEEM, the convolution kernel is used to extract the shallow information of the hazy image and passed to DFB. DFB focuses on haze and high-frequency information. This network connects three DFBs in series. As the network gradually deepens, a large amount of detailed information will be lost. Therefore, the features extracted by the three DFBs are connected in the channel direction to realize the fusion of deep and shallow information and preserve more details while retaining rich features. The fusion information obtained from FEEM is transferred to FFEM. First, CA and PA are used to further flexibly adjust the obtained fusion haze density information. Secondly, CEM is used to enhance context information and suppress redundant information. Then, the haze density image h(x) is obtained through convolution reconstruction. Finally, Equation (4) is used to obtain a clear haze-free image.

### 3.3. DFB Mathematical Model

**Figure 2:**
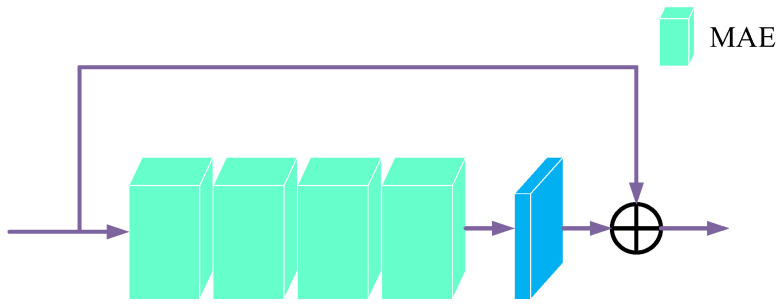
Structure of the DFB module.

DFB consists of multilevel attention enhancement module (MAE), convolutional layers and skip connections, as shown in Figure 2. MAE is introduced in Section 3.4. We use four consecutive MAE modules in DFB, which not only increases the network depth and expression to improve dehazing performance, but also ensures that the network’s parameter size remains relatively low. The skip connection prevents the gradient from disappearing as the depth increases and speeds up the training. The mathematical model of DFB is expressed as Equation (5).

(5) $$F_{D}^{*}=F_{D}+conv\left(\mathrm{MAE}\left(\mathrm{MAE}\left(\mathrm{MAE}\left(\mathrm{MAE}\left(F_{D}\right)\right)\right)\right)\right)$$

where $F_{D}$ is the input of DFB, $F_{D}^{*}$ is the output of DFB.

### 3.4. MAE Module Mathematical Model

**Figure 3:**
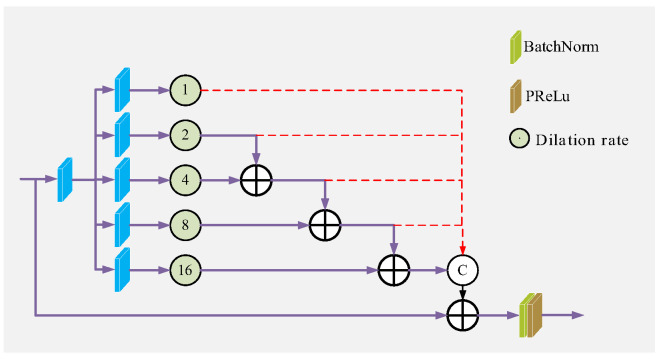
Structure of the SPDC.

**Figure 4:**
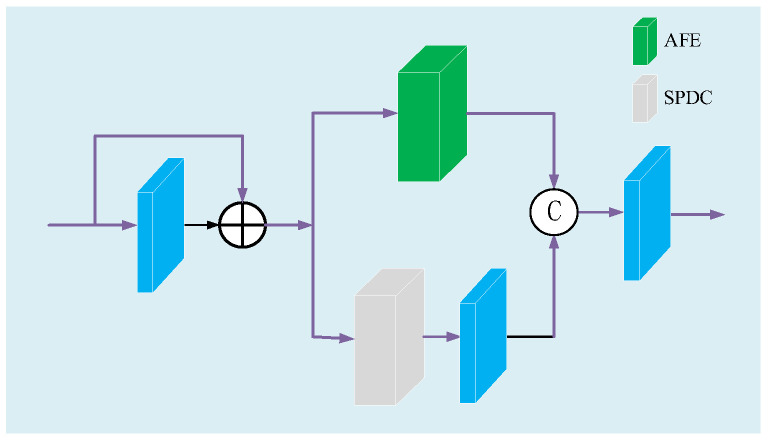
Structure of the MAE module.

Image features of different scales have different semantic information, so fully extracting and utilizing multi-scale features can effectively improve the quality of dehazing images. Compared with using different ordinary convolution kernels to adjust the size of the receptive field, dilated convolution can achieve the same performance without introducing other calculations and parameters. Mehta et al. [36] proposed the spatial pyramid of dilated convolutions (SPDC), as shown in Figure 3, which can eliminate the grid artifacts well by learning fewer parameters. Although the attention feature enhancement module (AFE) in Section 3.5 adopts the CEM module to improve the receptive field of the network, when the data stream is transmitted in the AFE module, some information will still be lost. Therefore, we propose the MAE module, as shown in Figure 4, to further reduce the loss of detailed information.

The input feature map passes through a local residual block in MAE and is then transmitted in parallel to the AFE module and the SPDC. The feature maps obtained from the two paths are concatenated and output. The multi-scale feature information collected by SPDC is a further supplement to the information obtained by the AFE module, which reduces the loss of haze information and high-frequency information during the transmission of the data stream through the AFE module, and eliminates grid artifacts. The MAE module mathematical model is shown in formula (6).

(6) $$F_{M}^{*}=conv\left(cat\left(\begin{array}{l} \mathrm{AFE}\left(F_{M}+conv\left(F_{M}\right)\right),\\ conv\left(\mathrm{SPDC}\left(F_{M}+conv\left(F_{M}\right)\right)\right) \end{array}\right)\right)$$

where $F_{M}$ is the input of the MAE module, $F_{M}^{*}$ is the output of the MAE module.

### 3.5. AFE Module Mathematical Model

**Figure 5:**
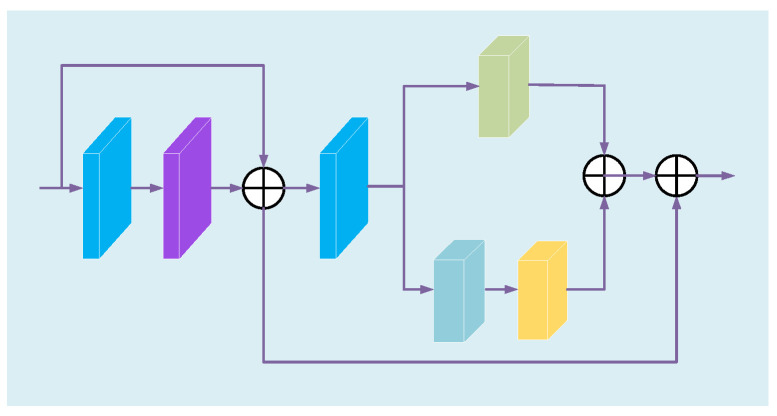
Structure of the AFE module.

In order to make up for the loss of some details in the existing attention module during image processing, we propose the AFE module, which is mainly composed of a local residual block, CA, PA and CEM, and the structure is shown in Figure 5. In AFE, local residual learning can avoid haze and low-frequency areas, and focus on effective information. CA uses maximum pooling and average pooling to aggregate channel information and fuse them, and adaptively assigns the weight of fusion information. In CA, inspired by Woo et al. [37], we improve the traditional channel attention mechanism, while using average pooling and max pooling to obtain finer channel attention and retain more detailed information of the image. PA [30] can flexibly focus on dense haze and high-frequency information, and has high sensitivity to images with uneven haze distribution. Then, we use the CEM module to increase the receptive field, improve the relevance of contextual information, and further obtain more detailed information. The AFE module mathematical model is shown by Equation (7).

(7) $$\begin{array}{cc} F_{A}^{*} & =\delta \left(conv\left(F_{A}\right)\right)+CA\left(conv\left(F_{A}+\delta \left(conv\left(F_{A}\right)\right)\right)\right)\\ & +CEM\left(PA\left(conv\left(F_{A}+\delta \left(conv\left(F_{A}\right)\right)\right)\right)\right) \end{array}$$

where $F_{A}$ is the input of the AFE module, $F_{A}^{*}$ is the output of the AFE module, δ is the Relu nonlinear function.

Our improved CA is shown in Figure 1. We combine the average pooling mechanism and the maximum pooling mechanism in parallel to obtain different channel features of the image in two ways and then fuse them to preserve more detailed features. Firstly, we aggregate the spatial information of the feature map using average pooling and max pooling to obtain different channel descriptors. Average pooling and max pooling are represented by Equations (8) and (9), respectively.

(8) $$g_{a}=H_{a}\left(F_{c}\right)=\frac{1}{H\times W}\sum_{i=1}^{H}\sum_{j=1}^{w}X_{c}\left(i,j\right)$$

(9) $$g_{m}=H_{m}\left(F_{c}\right)=Max\left(X_{c}\left(i,j\right)\right)$$

where $F_{c}$ is the input feature map, $X_{c}\left(i,j\right)$ is the value of the c-th channel $X_{c}$ at the (i,j) position, $H_{a}$ is the average function, $H_{m}$ is the maximum function.

Secondly, in order to focus on more image details, two convolutional layers and a Rule activation function are used to fuse the channel features on the two paths. The fusion function is expressed as:

(10) $$C_{f}=conv\left(\delta \left(conv\left(g_{a}\left(F_{c}\right)\right)\right)\right)+conv\left(\delta \left(conv\left(g_{m}\left(F_{c}\right)\right)\right)\right)$$

where $C_{f}$ is the fused channel feature. δ is the Relu function.

Then, we use the sigmoid function to assign the weights of different channels, the expression is as Equation (11).

(11) $$A_{c}=\sigma \left(C_{f}\right)$$

where $A_{c}$ is the weight value of the fusion feature, σ is the sigmoid function.

Finally, the input feature map $F_{c}$ is element-wise multiplied by the weight value $A_{c}$ to obtain the output result $F_{c}^{*}$, as shown by Equation (12).

(12) $$F_{c}^{*}=F_{c}⊗A_{c}$$

**Figure 6:**
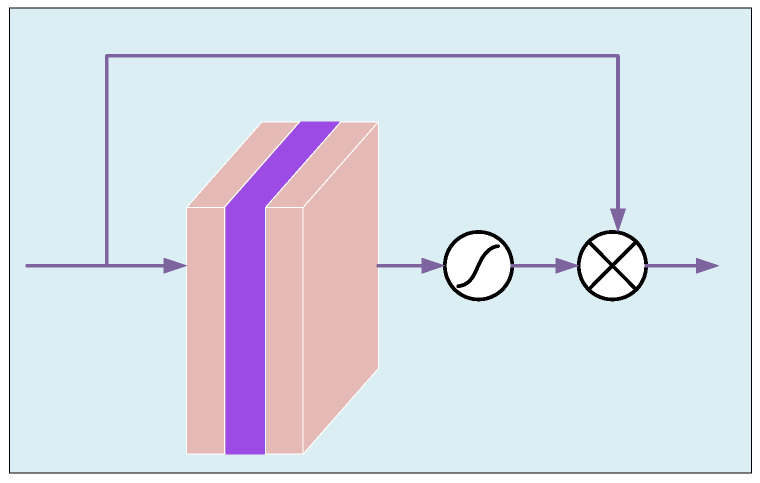
Structure of PA.

PA module can pay attention to thick-hazed pixels and high-frequency image region. As is shown in Figure 6, It contains two 1×1 convolutional layers, Relu and sigmoid activation function. The shape changes from C×H×W to 1×H×W. PA is shown by Equation (13)

(13) $$PA=\sigma \left(conv\left(\delta \left(conv\left(F_{PA}^{*}\right)\right)\right)\right)$$

where $F_{PA}^{*}$ is the input feature map of PA. Finally, $F_{PA}^{*}$ and PA are multiplied element-wise to obtain the output $F_{PA}$ of PA.

(14) $$F_{PA}=F_{PA}^{*}⊗PA$$

### 3.6. Context Enhancement Module Mathematical Model

Although PA can focus on high-frequency information, the receptive field of PA in shallow features is limited, making it difficult to ensure that subsequent feature learning has sufficient context information. In order to capture more context information, we use CEM based on the enhanced attention mechanism, as is shown Figure 1. The CEM module mathematical model is shown by Equations (15)–(17).

(15) $$f=conv\left(\overset{\vee }{K}\right)$$

(16) K∧=up(conv(Relu(conv(Relu(conv(max(conv(f))))))))

(17) $$K==\overset{\vee }{K}⊗\sigma \left(conv\left(conv\left(conv\left(\overset{\vee }{K}\right)\right)⊕\overset{\wedge }{K}\right)\right)$$

where $\overset{\vee }{K}$ and K are the input and output of the CEM module, respectively. f is the output of input $\overset{\vee }{K}$ through a 1×1 conv, $\overset{\wedge }{K}$ is the output of six consecutive operations of a 3×3conv with a step size of 2, Max Pooling, two consecutive 3×3conv with a step size of 1 and the Relu activation function, a 3×3conv with a step size of 1 and Upsample. Max Pooling is used to increase the receptive field, enhance contextual semantic information, and reduce redundant information. Its convolution kernel size is 7 and the step size is 3. max is Max Pooling, and up is Upsample.

### 3.7. Loss Function

To normalize the learning direction of our proposed network during training, we use the combination of $L_{1}$ loss and perceptual loss as the loss function for the whole training process. The $L_{1}$ loss is used to correct the difference of pixels between images, and the perceptual loss is used to normalize the human visual perception effect. The total loss function is expressed by Equation (18):

(18) $$Loss=\lambda_{1}L_{1}+\lambda_{p}L_{p}$$

where $\lambda_{1}$ and $\lambda_{p}$ are the weights of $L_{1}$ loss and perceptual loss, respectively. In this paper, we use the $L_{1}$ function as the main loss function. The perceptual loss function fine-tunes the dehazed image and the clear image at the depth feature level. According to the training experience, the values of $\lambda_{1}$ and $\lambda_{p}$ are set to 1 and 0.04, respectively.

#### 3.7.1. $L_{1}$ Loss

The $L_{1}$ loss function, also known as the mean absolute value error, is a regression model that compares differences pixel by pixel and takes the absolute value. Its mathematical expression is shown by Equation (19)

(19) $$L_{1}=\frac{1}{3N}\sum_{n=1}^{N}\sum_{c=1}^{3}\left|I_{c}\left(n\right)-I_{c}^{gt}\left(n\right)\right|$$

where N is the total number of pixels, c is the number of channels, $I_{c}\left(n\right)$ is the dehazed image restored by the network, and $I_{c}^{gt}\left(n\right)$ is the original image without haze. The $L_{1}$ function will create sparse features, reset the weight of useless features to 0, and have a feature selection effect.

#### 3.7.2. Perceptual Loss

In image restoration tasks, perceptual loss is widely applied to the perceptual difference between two images, which can obtain extremely rich, detailed information. In this paper, VGG-16 [38] is used as the pre-training network for perceptual loss, and the perceptual loss is calculated by the feature map output by VGG-16, which can be expressed as

(20) $$L_{p}=\sum_{i=1}^{3}\frac{1}{C_{j}H_{j}W_{j}}\left\|\varphi_{j}\left(J\right)-\varphi_{j}\left(J_{dehaze}\right)\right\|$$

where $\varphi_{j}$ is the feature map of layer j in the VGG-16 network, the size is $C_{j}\times H_{j}\times W_{j}$, J is the clear image, and $J_{dehaze}$ is the dehazed image.

## 4. Experimental Results

In this section, first we introduce the dataset and experimental details. The performance of our proposed method is then evaluated on synthetic and real-world datasets and compared with state-of-the-art dehazing methods. Finally, the effectiveness of our proposed module is demonstrated through ablation studies.

### 4.1. Datasets

We conducted experiments on the publicly available Realistic Single Image Dehazing(RESIDE) dataset [39]. The dataset consists of five subsets, including two training sets and three testing sets. The training sets are the indoor training set (ITS) and the outdoor training set (OTS); the testing sets are the synthetic objective testing set (SOTS), the hybrid subjective testing set (HSTS), and the real-world task-driven testing set (RTTS). For the train, we selected the OTS as the main training set, which contains 8970 clear outdoor images, each of which is combined with 35 hazy images, resulting in a total of 313,950 synthetic hazy images. To further verify the dehazing performance in real scenes, the real-world NHHAZE dataset [40] was also selected for training. For the test, the SOTS-outdoor are used for tests of synthetic hazy images (SOTS includes SOTS-indoor and SOTS-outdoor datasets), while the NHHAZE and RTTS datasets are used for testing real hazy images.

### 4.2. Implementation Details

We employed the Pytorch framework with NVIDIA RTX8000 GPU on both training and testing stages. The proposed network was trained in RGB channels and augment the training dataset randomly rotated by 90, 180, 270 degrees and horizontal flip. Images were resized to 240 × 240 through preprocessing, and the ADAM optimizer was implemented with a batch size of 4. The whole network is trained for $5\times 10^{5}$ steps on the OTS training set and NHHAZE training set.

The initial learning rate is set to 0.0001, we adopt the cosine annealing strategy [41] to adjust the learning rate from the initial value to 0 by following the cosine function. Expressly, we assume the total step is τ and the initial learning rate is η, the learning rate $\eta_{s}$ will be updated adaptively by the following strategy.

(21) $$\eta_{s}=\frac{1}{2}\left(1+\cos\left(\frac{\tau_{s}\pi }{\tau }\right)\right)\eta$$

**Figure 7:**
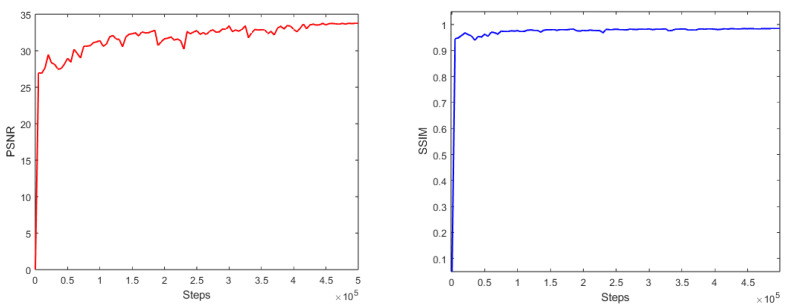
PSNR and SSIM learning curves. The red line and blue line are the change curves of PSNR and SSIM respectively as steps increase.

We train continuously and plot the learning curves by obtaining PSNR and SSIM scores every 5000 steps, as shown in Figure 7. We use skip connections to prevent the gradient from disappearing. When training for $5\times 10^{5}$ steps, PSNR and SSIM level off, and we stop learning. When training for $4.45\times 10^{5}$ steps, the PSNR score and SSIM score reach the maximum, and we select the model at this time as our best model.

### 4.3. Experimental Results on Synthetic Hazy Images

In this section, we compare our proposed architecture with the following state-of-the-art dehazing methods: DCP [16], CAP [17], AOD-Net [26], Dehaze-Net [24], FFA-Net [30], and GridDehaze-Net [31].

We use Peak Signal-to-Noise Ratio (PSNR), Structural Similarity (SSIM) and Learned Perceptual Image Patch Similarity (LPIPS) among image quality assessment methods to evaluate the dehazing performance of other state-of-the-art methods. The objective results of various dehazing methods on the synthetic dataset are shown in Table 1. It can be observed that the proposed method achieves the best dehazing performance compared to other methods, with PSNR and SSIM scores of 33.74 and 0.9843 on SOTS-outdoor. The LPIPS distance is only 0.0040. This objectively proves the dehazing advantage of the proposed method on synthetic hazy images. Moreover, our proposed method has fewer parameters (params) and floating point operations (Flops). Compared with FFA-Net, our method has $1.39\times 10^{6}$ fewer params and $331.55\times 10^{9}$ fewer Flops, but achieves better dehazing performance.

**Figure 8:**
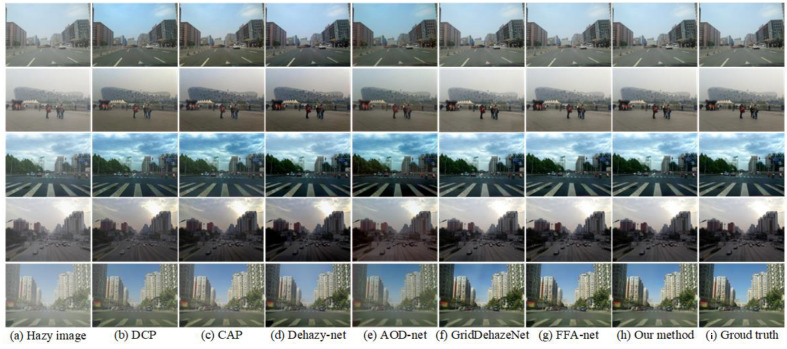
Visual results comparison on SOTS-outdoor dataset.

The subjective results of various dehazing methods on the SOTS-outdoor test set are shown in Figure 8. It can be found that compared with the ground truth image, CAP, DCP and Dehaze-net obviously dehaze excessively, such as the road surface in the first row of Figure 8b–d. AOD-net has the phenomenon of color distortion and dehazing image blur, as shown in the fourth and fifth rows of Figure 8e. GridDehaze-Net and FFA-Net achieve results that are closer to the ground truth images, as shown in Figure 8f,g. However, upon closer inspection, it can be observed that GridDehaze-Net’s and FFA-Net’s dehazed results exhibit subtle haze residue and are generally whiter than the ground truth image, as shown in the ground in the first and second rows of Figure 8f,g. The proposed method captures more detailed information in the images, and the dehazed results are most similar to the ground truth images.

### 4.4. Experimental Results on Real-World Hazy Images

**Figure 9:**
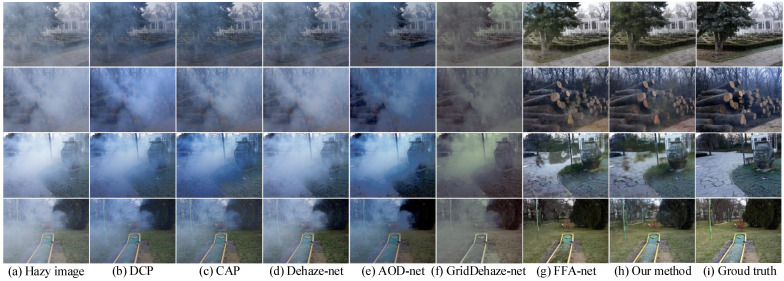
Visual results comparison on NHHAZE dataset.

To verify the dehazing performance of proposed method on real hazy images, we conducted tests on the NHHAZE and RTTS datasets, respectively. The subjective results on the NHHAZE dataset are shown in Figure 9. We can find that the effect of haze removal is not obvious for DCP, CAP, Dehaze-Net, and AOD-Net methods, with a large amount of hazy remaining and color distortion. GridDehaze-Net has obvious color distortion. Both FFA-net and our method have achieved a better dehazing effect. However, compared with the proposed method in this paper, FFA-net has more hazy residue, such as the upper left corner of the trees in the first row of Figure 9g. Our method removes haze obviously and retains detailed information such as color that is closer to the real image on the ground.

We also performed quantitative evaluations on the real hazy images in NHHAZE, as shown in Table 2, to demonstrate the effectiveness of our method further objectively for dehazing. It can be observed that in four randomly selected real hazy images in NHHAZE, our method achieves significantly better PSNR and SSIM scores compared with other dehazing methods. For example, in the first image of Table 2, the PSNR scores are improved by 2.49 and the SSIM scores is improved by 0.0023 compared with the FFA-net, which has the second highest dehazing performance. LPIPS distance is also the shortest among our methods; for example, it is only 0.159 in the fourth row of Table 2.

**Figure 10:**
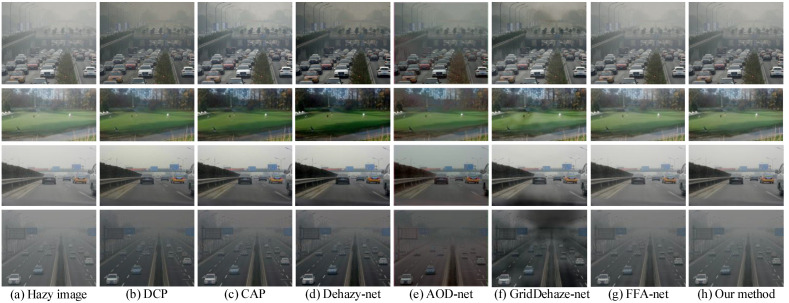
Visual results comparison on RTTS dataset.

The subjective results of various dehazing methods on the RTTS dataset are shown in Figure 10 (since the RTTS does not have ground truth images, only qualitative analysis was performed). We can know that DCP, CAP and Dehaze-Net are over-dehazing, the image is overall dark, and there is obvious color distortion, such as the road surface in the third row of Figure 10b–d. AOD-Net exhibits a color cast phenomenon, as shown in the fourth row of Figure 10e. GridDehaze-Net exhibits an uneven dehazing effect and loses the color information of the image, such as the fourth row of Figure 10f. FFA-net leaves a lot of haze, and the dehazing effect is not obvious, as shown in the lawn in the second row of Figure 10g. Our proposed method has a better color fidelity while having a clear dehazing effect.

**Figure 11:**
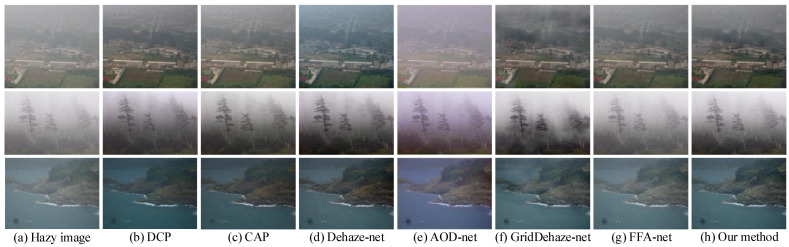
Visual results comparison on real-world images.

The subjective results of various dehazing methods in the real world are shown in Figure 11. We can know that DCP and CAP have obvious excessive dehazing phenomenon, such as the reef in the third row of Figure 11b,c. Dehaze-net and AOD-net have obvious color cast phenomena, such as the first rows of Figure 11d,e. GridDehaze-net only performs local dehazing and loses the texture information of the image, such as the upper right corner of the first row and the tree in the second row of Figure 11f. The dehazing effect of FFA-net is not obvious, as shown in the third line of Figure 11g. Our method better preserves the color and texture information of the image.

### 4.5. Ablation Study

To demonstrate the effectiveness of our proposed method, we conducted an ablation study to analyze the MAE and AFE modules. We cropped the image to 96×96 as input with training of $2\times 10^{5}$ steps; other configurations are the same as our implementation details. The quantitative evaluation results of different modules on the SOTS-outdoor dataset are shown in Table 3.

First, we constructed a baseline network without the SPDC and CEM moules, represented as “Baseline” in Table 3. Then, we added the SPDC module and CEM module separately to the baseline network, represented as “Baseline+SPDC” and “Baseline+CEM” in Table 3, respectively. Finally, we added both the SPDC and CEM modules to the baseline network, which is our proposed network model, represented as “Baseline+SPDC+CEM” in Table 3.

According to the quantitative results of the ablation study on the SOTS-outdoor dataset in Table 3, we can draw the following conclusions:

(1) When our proposed method does not include the SPDC and CEM modules, that is, the baseline network, the dehazing results are the worst.
(2) When the SPDC module is added to the Baseline network, compared with the dehazing results of the Baseline network, the PSNR scores is increased by 2.26, the SSIM scores is increased by 0.0152, and the LPIPS distance decreased by 0.0028, which proves the effectiveness of the SPDC module in improving the dehazing performance.
(3) When adding the CEM module on the baseline network, that is, the AFE module we proposed, compared with the dehazing results of the baseline network, the PSNR scores is increased by 2.48, the SSIM scores is increased by 0.0184, and the LPIPS distance decreased by 0.0028, which proves the dehazing performance of the proposed AFE module.
(4) When both SPDC module and CEM module are added to baseline network, that is, the MAE module proposed in this paper, the network model is our proposed method. The PSNR and SSIM scores are the highest and the LPIPS distance is the shortest in Table 3, indicating that our proposed MAE module and method have the best dehazing performance, demonstrating the superiority of our proposed MAE module and method.

## 5. Discussion

We developed a new image dehazing network model that demonstrated good dehazing performance. The MAE module and AFE module we proposed use the attention mechanism to adaptively extract haze features, the CEM module enhances contextual information, suppresses redundant information, and dilated convolution expands the receptive field, which can greatly compensate for the loss of detailed information. Therefore, compared with several other state-of-the-art dehazing algorithms such as AOD-net and FFA-net, our network can better capture the color, texture, and other detailed information of the image. Networks such as AOD-net optimize their network models by calculating mean square error (MSE). Our network is the same as FFA-net, using both PSNR and SSIM to optimize the network model. Therefore, our network can directly obtain the best network model corresponding to the maximum PSNR score and SSIM score. In addition, our proposed MAE module and AFE module are universal, which means that they can be easily plugged into network models in other fields. For example, in the field of medical imaging [42], they can retain more detailed information in images, helping doctors to judge the condition more accurately. Our network currently shows good performance in dehazing single images, but has not tested the dehazing performance on hazy video images. In the future, we hope to further expand our method to areas such as video dehazing to achieve real-time recovery of hazy videos.

## 6. Conclusions

In this paper, we propose an end-to-end multi-scale attention feature enhancement network for single image dehazing, which can well preserve image color, texture, and other detailed information. Around the goal of retaining more detailed information of images, we designed the Attention Feature Enhancement module and the Multi-Scale Attention Enhancement module, which focuses on high-frequency information and haze information. Our proposed network was tested on synthetic and real haze datasets for both qualitative and quantitative evaluation. Experimental results have shown that the proposed method has achieved state-of-the-art results. Through ablation studies, we studied the effectiveness of the different modules proposed.

## Figures and Tables

**Table 1:** Quantitative comparisons on synthetic datasets.

Method	SOTS-Outdoor			$\mathrm{Params}/\times 10^{6}$	$\mathrm{Flops}/\times 10^{9}$
	PSNR	SSIM	LPIPS		
DCP	21.81	0.8583	0.0527	—	—
CAP	22.09	0.8829	0.0427	—	—
AOD-Net	20.29	0.8765	0.0880	0.002	0.101
Dehaze-Net	22.46	0.8514	0.0390	0.008	0.450
GridDehaze-Net	30.86	0.9819	0.0053	0.956	452.01
FFA-Net	33.38	0.9804	0.0049	4.456	1010.86
Ours	33.74	0.9843	0.0040	3.070	679.31

**Table 2:** Quantitative comparisons on NHHAZE dataset (PSNR/SSIM/LPIPS).

Image No.	DCP	CAP	Dehaze-Net	AOD-Net	GridDehaze-Net	FFA-Net	Our Method
1	13.36/0.4838/0.386	13.52/0.4881/0.396	12.48/0.4715/0.410	13.38/0.4589/0.439	12.77/0.4854/0.413	18.65/0.6605/0.217	21.14/0.6628/0.213
2	11.83/0.4087/0.492	12.62/0.4060/0.498	11.16/0.3726/0.534	14.56/0.4253/0.537	12.13/0.4005/0.497	18.86/0.6109/0.288	20.88/0.6261/0.282
3	11.69/0.4977/0.460	12.10/0.4994/0.448	10.77/0.4882/0.477	11.50/0.4613/0.475	10.42/0.5044/0.446	17.00/0.6621/0.264	18.09/0.6647/0.260
4	13.84/0.5359/0.345	14.15/0.5303/0.342	12.94/0.5231/0.357	14.27/0.4562/0.434	13.65/0.5416/0.385	19.85/0.7259/0.167	22.34/0.7282/0.159

**Table 3:** Ablation study on SOTS-outdoor dateset.

Method	PSNR	SSIM	Lpips
Baseline	26.71	0.9514	0.0269
Baseline + SPDC	28.97	0.9666	0.0241
Baseline + CEM	29.19	0.9698	0.0241
Baseline + SPDC + CEM	29.50	0.9706	0.0232

## Data Availability

The RESIDE dataset and NH-HAZE dataset are made publicly available for research purposes. For more information, please refer to the websites https://sites.google.com/site/boyilics/website-builder/reside/, and https://data.vision.ee.ethz.ch/cvl/ntire20//nh-haze/ (accessed on 18 December 2022).
